# MicroRNAs in diabetic cardiomyopathy and clinical perspectives

**DOI:** 10.3389/fgene.2014.00185

**Published:** 2014-06-25

**Authors:** Qiulian Zhou, Dongchao Lv, Ping Chen, Tianzhao Xu, Siyi Fu, Jin Li, Yihua Bei

**Affiliations:** ^1^Regeneration Lab and Experimental Center of Life Sciences, School of Life Science, Shanghai UniversityShanghai, China; ^2^Shanghai Key Laboratory of Bio-Energy Crops, School of Life Science, Shanghai UniversityShanghai, China; ^3^Innovative Drug Research Center of Shanghai UniversityShanghai, China

**Keywords:** microRNAs, diabetes, cardiomyopathy, heart failure, biomarker

Diabetes is a progressive metabolic disorder that can ultimately lead to serious chronic vascular complications including renal failure, vision loss, and cardiac dysfunction (Ruiz and Chakrabarti, 2013). Diabetic cardiomyopathy is responsible for higher incidence of sudden cardiac death and represents the leading cause of morbidity and mortality among the diabetic patients (Aksnes et al., 2007; Chavali et al., 2013). Previous studies have indicated that oxidative stress and mitochondrial dysfunction were critically involved in the etiology of diabetes-induced cardiac dysfunction (Sugamura and Keaney, 2011; Styskal et al., 2012), that could subsequently induce a cascade of complex pathophysiological events characterized by early impairments of diastolic function, development of cardiomyocyte hypertrophy, myocardial fibrosis and cardiomyocyte apoptosis, eventually leading to heart failure (Huynh et al., 2014). However, the underlying mechanisms of diabetic cardiomyopathy are far from understood and current therapeutic strategies do not specifically aim at diabetic cardiomyopathy and diabetes-induced heart failure.

MicroRNAs (miRNAs, miRs), a novel class of non-coding RNAs of 22~24 nucleotides in length, act as post-transcriptional regulators of gene expression by binding to the 3′-untranslated region (3′-UTR) of target mRNA that induces mRNA degradation and/or translational repression (Lim et al., 2005; Van Rooij, 2011). Given that miRNAs are crucially involved in many critical biological processes including cell proliferation, apoptosis, necrosis, migration and differentiation (Bartel, 2004), desregulated miRNAs contribute to many human diseases including diabetes (Tyagi et al., 2011; Shantikumar et al., 2012; McClelland and Kantharidis, 2014) and cardiovascular diseases (Xiao et al., 2012; Fu et al., 2013; Vickers et al., 2014). Recent studies demonstrate that aberrant expression of miRNAs also participates in the pathogenetic processes mediating diabetic cardiomyopathy, where miR-1, -133, -141, -206, -223 have been reported upregulated, whereas miR-133a, -373, and -499 downregulated (Shen et al., 2011; Shantikumar et al., 2012; Asrih and Steffens, 2013). Thus, it is of crucial importance to gain insight into the role of miRNAs in the development of diabetic cardiomyopathy which will help clarify the molecular mechanisms as well as identify novel therapeutic strategies for diabetic cardiomyopathy.

Cardiomyocyte hypertrophy, myocardial fibrosis, and cardiomyocyte apoptosis are important features of diabetic cardiomyopathy (Ruiz and Chakrabarti, 2013). Downregulation of miR-133a induces cardiomyocyte hypertrophy via upregulating the expression of MEF2A and MEF2C, two transcription factors involved in myocardial hypertrophy (Feng et al., 2010). While upregulation of miR-1 and -206 contributes to increased cardiomyocyte apoptosis, via repressing the expression of heat shock protein (Hsp) 60, PIM 1, and IGF-1 receptor (Yu et al., 2008; Shan et al., 2010; Katare et al., 2011). In addition, miR-373 is downregulated in diabetic heart, which is supposed to induce cardiac fibrosis via regulating the expression of p300 (Feng et al., 2008; Chen et al., 2010; Shen et al., 2011; Chavali et al., 2014). Thus, these reports indicate the critical contribution of miRNAs in cardiomyocyte hypertrophy, myocardial fibrosis, and cardiomyocyte apoptosis during the development of diabetic cardiomyopathy.

Hyperglycemia, oxidative stress and mitochondrial damage are involved in the etiology of diabetes-induced cardiac dysfunction and diabetic cardiomyopathy (Shantikumar et al., 2012; Asrih and Steffens, 2013; McClelland and Kantharidis, 2014). Previous study has shown that miR-499 and -133a were markedly downregulated in the diabetic cardiomyocytes, while normalization of oxidant/antioxidant level by the treatment of N-acetylcysteine (NAC) restored the impaired expression of these miRNAs, indicating that hyperglycemia-induced downregulation of miR-499 and -133a was oxidative stress dependent (Yildirim et al., 2013). Similarly, miR-373 was downregulated by hyperglycemia-induced oxidative stress in diabetic cardiomyopathy via p38 MAPK pathway (Shen et al., 2011). MiR-141, a critical regulator of the inner mitochondrial phosphate carrier (Slc25a3), has been shown upregulated in diabetic heart, thus leading to the impaired mitochondrial ATP production in the pathogenesis of diabetic cardiomyopathy (Baseler et al., 2012). In terms of cardiomyocyte glucose metabolism, miR-223 was shown to be upregulated in the left ventricular biopsies of diabetic patients, which induced Glut4 protein level in cardiomyocytes and contributed to cardiomyocyte glucose uptake *in vitro*, indicating that overexpression of miR-223 might be a compensatory response to restore glucose metabolism in diabetic heart (Lu et al., 2010).

Accumulating evidence has indicated that circulating miRNAs can be used as sensitive biomarkers for certain diseases such as cardiovascular diseases and cancers (Fabbri, 2010; Tijsen et al., 2012; Xu et al., 2012). Despite that diabetes is among the major risk factors for cardiovascular complications, researches investigating circulating miRNAs in diabetic patients are quite limited. Zampetaki et al. reported deregulation of 12 plasma miRNAs (miR-24, -21, -20b, -15a, -126, -191, -197, -223, -320, -486, -150, and -28-3p) in diabetic subjects, among which miR-126 emerged as a predictor of diabetes mellitus (Zampetaki et al., 2010). A separate study identified 7 upregulated serum miRNAs (miR-9, -29a, -30d, -34, -124, -146a, and -375) in newly diagnosed type 2 diabetic patients as compared to susceptible controls (Kong et al., 2011). Another study identified elevation of miR-144, 192, and 29a in the whole blood of diabetic patients (Karolina et al., 2011), whereas no change was found in miR-126 level which was inconsistent with the report published by Zampetaki et al. (2010). This may be explained by different biosamples detected (plasma vs. whole blood) in these two studies (Zampetaki et al., 2010; Karolina et al., 2011). In addition, miR-503 was found to be enriched in the plasma of diabetic patients with critical limb ischemia (Caporali et al., 2011). However, to date, no specific circulating miRNA has been identified in diabetic cardiomyopathy. The diagnostic and predicted value of circulating miRNAs as biomarkers for diabetic cardiomyopathy remains to be further explored.

Taken together, desregulated miRNAs are potentially involved in the etiology and pathogenetic processes of diabetic cardiomyopathy. An in-depth understanding of their functional roles and molecular mechanisms in the development of diabetic cardiomyopathy will provide better prospects to identify sensitive clinical biomarkers and novel therapeutic targets for diabetic cardiomyopathy.

## Conflict of interest statement

The authors declare that the research was conducted in the absence of any commercial or financial relationships that could be construed as a potential conflict of interest.
